# Retraction: MiR-942 regulates the function of breast cancer cell by targeting FOXA2

**DOI:** 10.1042/BSR-2019-2298_RET

**Published:** 2023-02-10

**Keywords:** Breast cancer, FOXA2, MicroRNA-942, Prognosis, Progression

This Retraction follows an Expression of Concern relating to this article previously published by Portland Press. This article is being retracted from *Bioscience Reports* at the request of the Editor-in-Chief and the Editorial Board following receipt of a notification from a reader, alerting the Editorial Board to concerns over the western blots, Kaplan-Meier graphs and cell images in the paper. Specifically, the western blots have concerning features such as the shapes of the bands and absence of any detectable background, and the Kaplan-Meier graphs in Figures 1B and 4J show implausible date ranges. The Figure 1K Inhibitor cell sample also seems to appear as the Figure 8E Fox01+mimic panel (flipped 180 degrees) from Guo et al. 2020 (doi: 10.1186/s12935-020-01332-6). The authors have been contacted with regards to the retraction and have not responded to the Journal's queries or the concerns raised. Given the extent of the issues raised, the Editorial Board stand by the decision to retract the article.

